# Electrochemical Sensors Based on Au Nanoparticles Decorated Pyrene-Reduced Graphene Oxide for Hydrazine, 4-Nitrophenol and Hg^2+^ Detection in Water

**DOI:** 10.3390/molecules27238490

**Published:** 2022-12-02

**Authors:** Alma Mejri, Giacomo Mandriota, Hamza Elfil, Maria Lucia Curri, Chiara Ingrosso, Abdelmoneim Mars

**Affiliations:** 1 Laboratory of Natural Water Treatment (LADVEN), Water Researches and Technologies Center, University of Carthage, Techno-Park Borj-Cedria, BP 273, Soliman 8020, Tunisia; 2 CNR-IPCF Sez. Bari, c/o Department of Chemistry, Università degli Studi di Bari, Via Orabona 4, I-70126 Bari, Italy; 3 Department of Chemistry, Università degli Studi di Bari, Via Orabona 4, I-70126 Bari, Italy

**Keywords:** reduced graphene oxide, colloidal Au nanoparticles, nanocomposite, Hg^2+^ ion imprinted polycurcumin, electrochemical sensor, hydrazine, 4-nitrophenol, Hg^2+^, real water samples

## Abstract

Monitoring hazardous chemical compounds such as hydrazine (N_2_H_4_), 4-nitrophenol (4-NP) and Hg^2+^ in natural water resources is a crucial issue due to their toxic effects on human health and catastrophic impact on the environment. Electrochemical nanostructured platforms integrating hybrid nanocomposites based on graphene derivatives and inorganic nanoparticles (NPs) are of great interest for such a purpose. In this work, disposable screen-printed carbon electrodes (SPCEs) have been modified with a hybrid nanocomposite formed by reduced graphene oxide (RGO), functionalized by 1-pyrene carboxylic acid (PCA), and decorated by colloidal Au NPs. These hybrid platforms have been tested for the electrocatalytic detection of N_2_H_4_ and 4-NP by differential pulse voltammetry and have been modified with an electropolymerized film of Hg^2+^ ions imprinted polycurcumin for the electroanalytical detection of Hg^2+^ by DPV. LODs, lower and in line with the lowest ones reported for state-of-the-art electrochemical sensors, integrating similar Au-graphene < nanocomposites, have been estimated. Additionally, good repeatability, reproducibility, and storage stability have been assessed, as well as a high selectivity in the presence of a 100-fold higher concentration of interfering species. The applicability of the proposed platforms for the detection of the compounds in real complex matrices, such as tap and river water samples, has been effectively demonstrated.

## 1. Introduction

Population expansion, rapid urbanization, and intensive industrial activities with uncontrolled wastewater disposal are responsible for the production and discharge of high levels of hazardous compounds in water, harmfully affecting human health and the ecosystem. Among these, hydrazine (N_2_H_4_) is a strongly poisonous pollutant agent, soluble in water, that can be potentially adsorbed by human body tissues leading to blood, liver, and kidney disorders [1]. N_2_H_4_ is widely used in fuel cells, insecticides, photography, emulsifiers, chemicals, herbicides, and dyes, but also as a component of drugs [2,3]. U.S Environmental Protection Agency (U.S EPA) has defined N_2_H_4_ as a potent carcinogen, whose concentration threshold, in drinking water, is required to be lower than 10 ppb [4]. 4-nitrophenol (4-NP) is another dangerous contaminant highly stable, toxic, and water-soluble [5], used as an intermediate in pharmaceuticals, pesticides, and dyes production [6]. Even at trace levels, it causes damage to the human central nervous system, blood, liver, and kidneys [7], and it has been classified on the “Priority Pollutant List” by U.S. EPA, with an upper limit in drinking water of 10 ppb. Mercury (Hg) is a neurotoxic compound that can damage neurological, immune, enzyme, cardiovascular, respiratory, and gastrointestinal systems [8]. It can easily escape the cellular control mechanisms and bind protein, DNA, and nuclear proteins causing serious damage to cells [9]. Once introduced into the marine environment, bacteria convert inorganic Hg^2+^ into methylmercury, which can enter the food chain and accumulate in higher organisms [10]. U.S. EPA has set the Hg^2+^ limit at 3 ppb in drinking water [11].

To mitigate the effects of these dangerous species, many international organizations have undertaken actions aiming at developing sustainable and innovative technologies for their recognition and monitoring. In this perspective, highly sensitive and reproducible analytical methods such as atomic absorption spectrophotometry, high-performance liquid chromatography (HPLC), and inductively coupled plasma mass spectrometry (ICP), Ref. [12] have been developed. These approaches, however, usually require expensive, complex, and bulky instrumentation that cannot be used on-site, along with trained operators. Electrochemical sensors are valuable alternative methods for field use, real-time, and on-site detection of these compounds, due to their lightweight, portability, cost-effectiveness, and their limited demand in terms of electrical power supply [13]. The sensitivity of such sensors has been improved by implementing highly specific electrochemical (bio)sensing approaches, such as immunosensing, enzymatic sensing, molecularly imprinted polymers (MIP), or ion-imprinted polymers (IIP), and by taking advantage of nanostructures modified electrodes [14]. Nanostructured materials feature a high surface-active area, that along with their unique size and shape-dependent chemical–physical properties [15], make them particularly suited for transducing effective interactions with analytes, finally resulting in an enhanced sensitivity.

Graphene derivatives have found application in electrochemical sensors for their high conductivity, high electrocatalytic activity, remarkably fast heterogeneous electron transfer kinetics, Ref. [16] and high chemical reactivity, encouraging the manufacturing of a variety of functional hybrid materials with nanoparticles (NPs) and biomolecules. On the other hand, the use of Au NPs in electrochemical (bio)sensors is advantageous for their capability of binding biomolecules, high rate of heterogeneous electron transfer kinetics, and excellent surface redox electrocatalytic activity [17]. The combination of the graphene derivatives with Au NPs in hybrid nanocomposites empowers interphase electron coupling, yields materials with a very large electrochemically active surface area, and enhanced heterogeneous electron transfer kinetics, resulting in a rapid and sensitive current response [18,19,20]. Therefore, nanostructured platforms formed by these hybrid nanocomposites have been applied for the electroanalytical determination of a variety of analytes, including N_2_H_4_, 4-NP, and Hg^2+^ [21,22,23,24,25,26,27,28,29,30,31,32,33].

Disposable screen-printed carbon electrodes (SPCEs), modified by hybrid nanocomposites formed by reduced graphene oxide (rGO), functionalized with 1-pyrene carboxylic acid (PCA) and decorated by a dense layer of Au NPs (AuNPs/PCA-rGO/SPCEs), which have been recently demonstrated well-performing for the detection of a variety of analytes including cancer biomarker in human blood serum [18], vitamin C in food products [19] and As(III) in water [20], have been here investigated for the electrocatalytic detection of N_2_H_4_ and 4-NP, and after modification with Hg^2+^ ions imprinted polycurcumin (polyCM) films, for the electroanalytical quantification of Hg^2+^ by differential pulse voltammetry. 

CM, a polyphenol that presents in nature in two tautomeric forms (enol and keto), has been intensively applied in electroanalysis because it can detect simultaneously, cations and anions, that form, respectively, complexes with the keto tautomer form O,O′-donor binding sites, and hydrogen bonds with its enol hydroxyl groups. In addition, in CM, the two methoxy phenol and ketone groups can generate free radicals allowing its reproducible electropolymerization onto electrodes in stable films [34]. 

The electropolymerization is often performed in potentiodynamic mode by applying multiple cyclic voltammetry scans, which generate phenoxyl radicals undergoing oxidation to quinone or addition reactions in o- and p-positions, generating a poly(phenylene oxide) film [34]. PolyCM films, electropolymerized onto MnO_2_-Graphene nanosheets modified glassy carbon electrodes (GCEs), have been applied for the detection of Hg^2+^, along with fluoride and cyanide anions. The simultaneous binding of fluoride and cyanide ions results in a cathodic shift of the CM oxidation potential, whilst an anodic shift occurs for Hg^2+^ chelation [35]. When the electropolymerization of CM is performed in presence of Hg^2+^ ions, such species act as templating agents, as the CM monomers self-assemble around them, binding them by coordination, and after their leaching, cavities, and binding sites specific for Hg^2+^ ions are imprinted, conveying to the sensor platform a high selectivity and a high chemical binding affinity [36].

Here, the achieved hybrid modified platforms feature LODs lower or comparable with the lowest ones reported in the literature [21,22,23,24,25,26,27,28,29,30,31,32,33]. Repeatability, reproducibility, storage stability, and selectivity of the electrodes, manufactured for the detection of the selected hazardous compounds in real samples of the complex aqueous matrix from tap and river water, have been assessed, supporting their technological potential for monitoring environmental threats.

## 2. Results and Discussion

### 2.1. Synthesis and Characterization of the AuNPs/PCA-rGO Hybrid Nanocomposite

The AuNPs/PCA-rGO hybrid nanocomposite was synthesized according to the strategy reported in [37]; at first, commercial reduced graphene oxide (rGO) was exfoliated and surface functionalized with PCA by means of successive cycles of sonication, stirring, and centrifugation [37]. PCA demonstrated to act as a “molecular wedge” opening small gaps between the graphene sheet edges, penetrating deeper between the rGO multilayers, and anchoring to the rGO basal plane by *π–π* stacking interactions [37]. The out-of-plane -COOH functional groups resulted suited to keep separated the graphene sheets preventing their *π–π* re-stacking, and as coordinating sites of the formed colloidal Au NPs [37]. 

The morphology investigation performed by transmission electron microscopy (TEM) showed that the achieved PCA-rGO sheets are rather smooth and electron transparent, with higher image contrast area originating from folded edges and mechanical lattice deformations (Figure S1A of Supporting Material) [37].

The AuNPs/PCA-rGO hybrid nanocomposite was synthesized onto the PCA-rGO complex, by a two-step chemical reduction of the Au precursor in presence of DMBT, acting as the reducing, coordinating, and stabilizing agent, and of the reducing agent NaBH_4_. 

An aqueous solution of the Au precursor was added to a toluene solution of TOAB, where the dispersion of PCA-RGO in NMP was injected. Electrostatic interactions between TOAB and the Au precursor allowed the Au precursor transfer from the aqueous to the toluene phase, where PCA-RGO was dispersed, and reduction of Au(III) precursor to Au(I) was induced by DMBT, which coordinates the surface of the NPs controlling their growth and morphology, and finally to Au(0) by NaBH_4_ addition [37]. In such a process, the colloidal Au NPs heteronucleate and grow anchored onto the rGO basal plane, thanks to their coordination to the -COOH groups of PCA, and concomitantly homonucleate in the synthesis solution (Figure S1B) [37]. 

**Figure 1:**
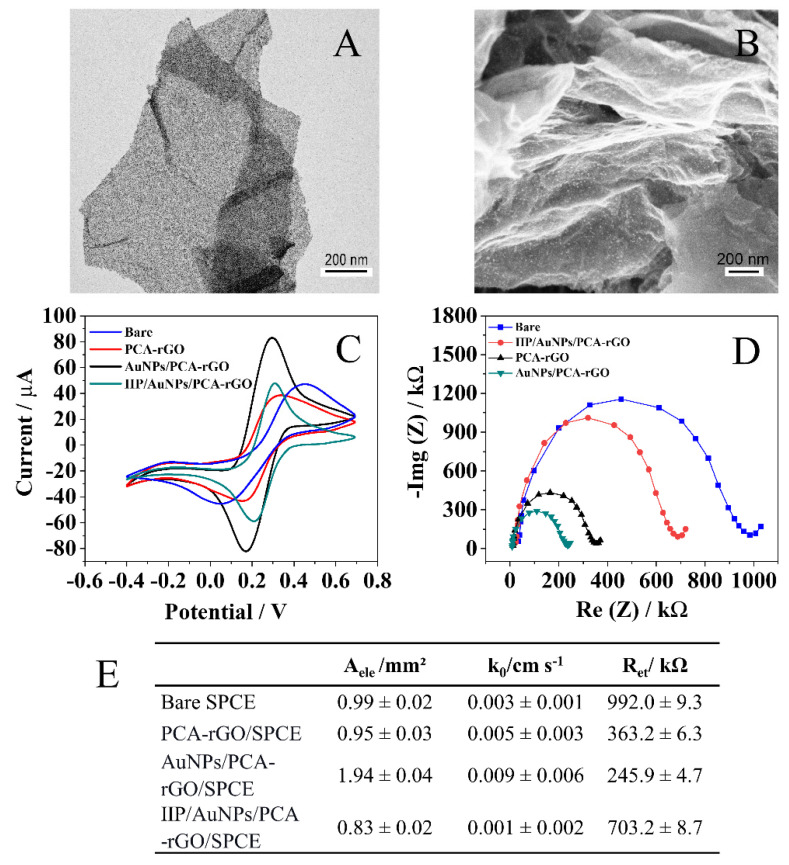
(**A**) TEM micrograph of the AuNPs/PCA-rGO and (**B**) SEM image of the AuNPs/PCA-rGO/SPCEs (127.92 KX). (**C**) CV scans collected at 50 mV s^−1^ and (**D**) EIS spectra of the SPCEs, PCA-rGO/SPCEs, AuNPs/PCA-rGO/SPCEs, and IIP/AuNPs/PCA-rGO/SPCEs, in 0.01 M PBS (pH 7.4), 5 mM in Fe[(CN)_6_]^3−/4−^ and 0.1 M in KCl. (**E**) Electroactive surface area (A_ele_), apparent heterogeneous electron transfer constant (K_0_), and electron transfer resistance (R_et_) of the SPCEs, PCA-rGO/SPCEs, AuNPs/PCA-rGO/SPCEs, and IIP/AuNPs/PCA-rGO/SPCEs.

The as-synthesized AuNPs/PCA-RGO sample was purified by addition of methanol in excess followed by centrifugation, and the effectiveness of such treatment was assessed in the TEM images by the lack of contamination from residual ligand molecules [38], as well as crystallites of the Au precursor, NaBH_4_, and TOAB (Figure 1A and Figure S1B).

Then, to separate the hybrid nanocomposite sheets from the homonucleated colloidal Au NPs, the separation strategy optimized in [37] was performed. The morphology of the isolated hybrid nanocomposite appears characterized by high image contrast, spherical nano-objects, 2–3 nm in size (Figure 1A and Figure S1C), that can be ascribed to the DMBT-coated Au NPs, binding the rGO sheets by means of *π–π* stacking interactions, and resulting in dense stacked layers, whilst the homonucleated NPs were collected in the supernatant (Figure S1D).

### 2.2. Electrochemical Characterization of the AuNPs/PCA-rGO Modified SPCEs 

SPCEs were modified with the AuNPs/PCA-rGO nanocomposite as reported in the Materials and Methods section. The manufactured hybrid AuNPs/PCA-rGO/SPCEs features the wrinkled sheet-like structures (Figure 1B) observed in the SEM images of the PCA-rGO/SPCEs (Figure S2A), and ascribed to the PCA-rGO complex, uniformly, and densely coated by nanometer-sized bright contrast DMBT-Au NPs (Figure 1B) [37].

The modification of the SPCEs with PCA-rGO and AuNPs/PCA-rGO, the latter both as neat and after in situ electrodeposition of Hg^2+^ ions imprinted polyCM (IIP) films, was assessed by cyclic voltammetry (CV) and electrochemical impedance spectroscopy (EIS) (Figure 1C,D), in presence of Fe[(CN)_6_]^3₋/4₋^, an inner-sphere electroactive redox probe very sensitive to the electrode surface chemistry and structure. 

The PCA-rGO/SPCEs and AuNPs/PCA-rGO/SPCEs show, in the presence of [Fe(CN)_6_]^3₋/4₋^, a decrease in the anodic and cathodic peak potentials (ΔEp) difference with respect to the bare SPCEs, which feature, instead, the couple of quasi reversible redox peaks typical of [Fe(CN)_6_]^3₋/4₋^ (Figure 1C). This result can be due to higher reversibility of the redox probe behavior at both the modified electrodes, which can be attributed to higher conductivity and an enhanced electron transfer capability at the electrode/electrolyte interface. This hypothesis is confirmed by the 1.7- and 3-fold enhancement of the apparent heterogeneous electron transfer kinetic constants K_0_ (Figure 1E) of the PCA-rGO/SPCEs and AuNPs/PCA-rGO/SPCEs, with respect to the bare SPCEs, estimated as reported in Equation (2) for a quasi-reversible system.

As far as the PCA-rGO/SPCEs, the increase in K_0_ can be reasonably explained by the PCA linker which can solder adjacent graphene sheets, ensuring their electric contact across the film deposited onto the electrode [39], and to the reactive -COOH and -OH groups available for undergoing red/ox reactions [40]. 

Moreover, the ΔEp decrease is more evident in the AuNPs/PCA-rGO/SPCEs compared to the PCA-rGO/SPCEs (Figure 1C), likely du e to i. Au NPs-rGO electron coupling phenomena occurring through PCA molecules and increasing the electrode conductivity, and ii. the presence of the Au NPs catalyzing the [Fe(CN)_6_]^3₋/4₋^ red/ox processes at the electrode surface [18,19,20,41].

Finally, the AuNPs/PCA-rGO/SPCEs show a remarkable increase in the current intensity with respect to the PCA-rGO/SPCEs (Figure 1C), attesting to a higher electrochemical reactivity, reasonably due to the two-fold increase in the electroactive surface area (A_ele_) (Figure 1E) estimated by the Randles–Sevcik equation (Equation (1)) [18,19,20]. 

The Nyquist plots show a considerable decrease in the semicircle diameter of the EIS spectra of AuNPs/PCA-rGO/SPCEs and PCA-rGO/SPCEs, with respect to the bare SPCEs (Figure 1D), confirming the higher reversibility of the redox probe at such electrodes, as assessed by the decrease in the electron transfer resistance (R_et_) estimated by Equation (2) (Figure 1E). 

CV scans and EIS curves show a different behavior of the IIP/AuNPs/PCA-rGO/SPCEs compared to the AuNPs/PCA-rGO/SPCEs (Figure 1C,D). Specifically, whilst ΔEp keeps almost the same value for both electrodes (Figure 1C), the peak current intensity (Figure 1C), and the semicircle diameter of the EIS spectra (Figure 1D) at the IIP/AuNPs/PCA-rGO/SPCEs are, respectively, lower, and higher with respect to those of the AuNPs/PCA-rGO/SPCEs, due to the lower A_ele_ and k_0_ (Figure 1E). These results can be reasonably explained by the blocking properties of the IIP film, limiting heterogeneous electron transfer kinetics at the electrode/electrolyte interphase [42].

### 2.3. Electroanalytical Application of the AuNPs/PCA-rGO/SPCEs

#### 2.3.1. Electrochemical Detection of N_2_H_4_ and 4-NP by the AuNPs/PCA-rGO/SPCEs

**Figure 2:**
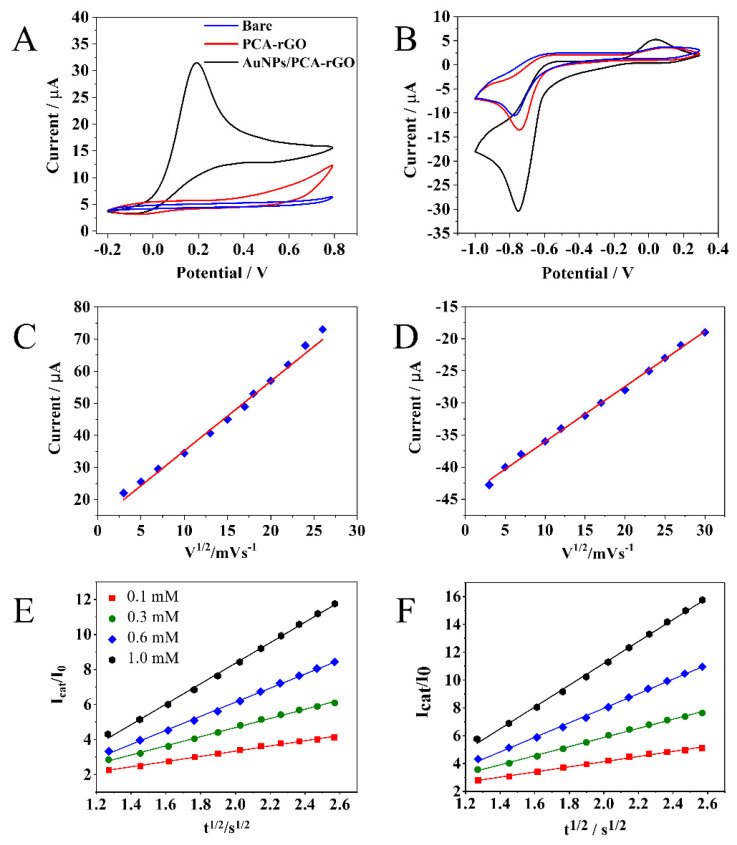
CV scans recorded at the SPCEs, PCA-rGO/SPCEs, and AuNPs/PCA-rGO/SPCEs, at 100 mV s^₋1^, in 0.1 mM PBS buffer (pH 7.4), (**A**) 1 mM in N_2_H_4_, from −0.2 V to 0.7 V, and (**B**) 1 mM in 4-NP, from −1 V to 0.3 V. Oxidation currents of N_2_H_4_ at +0.15 V (**C**,**E**) and reduction currents of 4-NP at −0.71 V (**D**,**F**) at the AuNPs/PCA-rGO/SPCEs, between 100–500 mV s^₋1^, versus square root of scan rate (**C**,**D**) and versus square root of time (**E**,**F**), in the 0.1–1 mM range in 0.1 M PBS buffer solutions (pH 7.4).

The electrochemical performance of the SPCEs, PCA-rGO/SPCEs, and AuNPs/PCA-rGO/SPCEs in the detection of 4-NP and N_2_H_4_ was investigated by collecting CV scans in PBS buffer solutions added by N_2_H_4_ (Figure 2A) and 4-NP (Figure 2B), respectively, keeping constant the electrode geometrical area (4 mm diameter, 12.6 mm^2^). 

In presence of N_2_H_4_, the CV scans recorded at the SPCEs and PCA-rGO/SPCEs do not show any peak, whilst the AuNPs/PCA-rGO/SPCEs present a large and intense signal at 0.19 V (vs. pseudo-Ag/AgCl) (Figure 2A), which is ascribed to N_2_H_4_ oxidation catalyzed by the Au NPs [43]. Such a process occurs with the formation of the transient radical cation N_2_H_4_^+^ at the electrode surface, which is then oxidized to N_2_ with the transfer of four electrons to the electrode and the release of four protons [44]. During the reverse scan, no cathodic peak is observed for N_2_H_4_ (Figure 2A), pointing out the irreversibility of the oxidation process.

In the presence of 4-NP, the CVs recorded at the SPCEs show a peak at ca. −0.77 V (vs. pseudo-Ag/AgCl) due to the reduction of 4-NP to 4-aminophenol [45] (Figure 2B). This peak positively shifts to −0.74 V (vs. pseudo-Ag/AgCl) at the PCA-rGO/SPCEs, and to −0.71 V (vs. pseudo-Ag/AgCl), at the AuNPs/PCA-rGO/SPCEs (Figure 2B), indicating that, both at the PCA-rGO/SPCEs and AuNPs/PCA-rGO/SPCEs, the reduction of 4-NP is more energetically favored than at the bare SPCEs, likely due to their higher electron conductivity and higher K_0_ (Figure 1E). 

The larger shift of the 4-NP reduction potential towards the lower energy at the AuNPs/PCA-rGO/SPCEs is due to the electrocatalytic activity of the Au NPs and to the PCA-induced Au-rGO electron coupling [18,19,20,41]. 

Finally, an increase in the current intensity of the reduction peak of 4-NP at the PCA-rGO/SPCEs and AuNPs/PCA-rGO/SPCEs, more evident at the hybrid electrodes was observed (Figure 2B), being reasonably due to the increase in the A_ele_ of the PCA-rGO/SPCEs and AuNPs/PCA-rGO/SPCEs (Figure 1E). It is worth noticing that the peak at ca. 0.03 V is accounted for by 4-quinoimine, which is the reduction product of 4-aminophenol [45], and it appears more pronounced at the AuNPs/PCA-rGO/SPCEs (Figure 2B) due the Au NPs catalyzing its oxidation [43].

To investigate the charge transport characteristics at the AuNPs/PCA-rGO/SPCEs and determine the mass transfer regime, the effect of the scan rate on the current intensity of N_2_H_4_ and 4-NP was studied (Figure 2C,D).

Figure 2C reports the N_2_H_4_ oxidation peak, at +0.15 V, as a function of the square root of the scan rate (v^1/2^). Such a plot can be fitted by linear regression with a correlation coefficient of 0.99, increasing linearly with the increase in v^1/2^ (Figure 2C) and the same trend can be observed for the reduction current of 4-NP (Figure 2D). These results indicate, in both cases, the occurrence of diffusion-controlled electron transfer processes.

Chronoamperometry measurements were performed to estimate the electrocatalytic rate constants (K_cat_) of the N_2_H_4_ oxidation and 4-NP reduction at the AuNPs/PCA-rGO/SPCEs. Figure 2E,F report the catalytic and initial current intensity ratio (I_cat_/I_0_) of N_2_H_4_ and 4-NP versus the square root of time (t^1/2^), respectively, in the concentration range of 0.1–1.0 mM. The plots show a linear relationship of I_cat_/I_0_ vs t^1/2^, and the mean Kcat values, estimated according to the Cottrell equation (Equation (3)), are 8.3 mM^₋1^ s^₋1^ for N_2_H_4_ and 6.9 mM^₋1^ s^₋1^ for 4-NP, indicating an excellent electrocatalytic activity of the AuNPs/PCA-rGO/SPCEs for the N_2_H_4_ oxidation and the 4-NP reduction.

##### Calibration Procedure, Repeatability, Reproducibility, and Storage Stability of AuNPs/PCA-rGO/SPCEs

**Figure 3:**
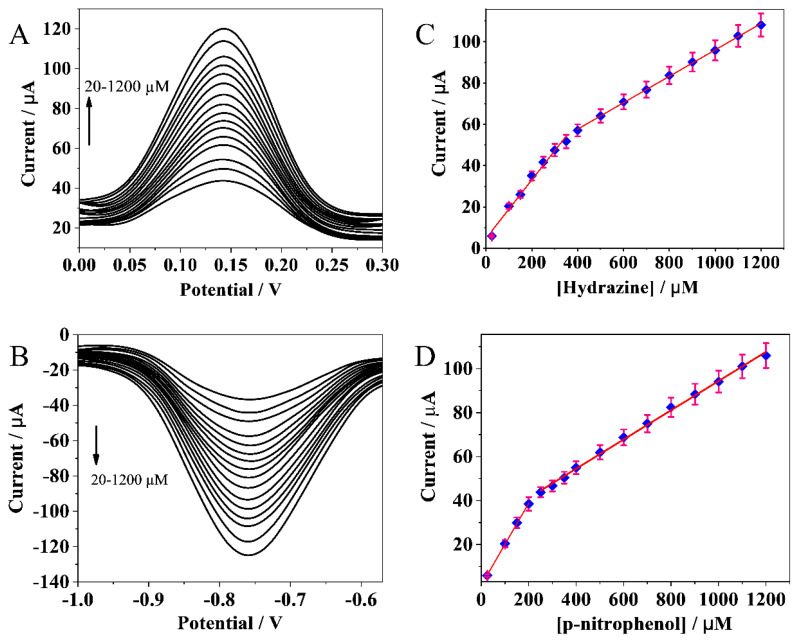
DPVs (**A**,**B**) and corresponding calibration plots (**C**,**D**) of N_2_H_4_ (**A**,**C**) and 4-NP (**B**,**D**) between 20–1200 µM, in 0.1 M PBS buffer (pH 7.4) at the AuNPs/PCA-rGO/SPCEs, with 0.05 s modulation time, 0.2 s interval time, 60 mV modulation amplitude, 10.5 mV step potential, and 50 mV s^₋1^ scan rate.

Differential pulse voltammetry (DPV) measurements were performed at the AuNPs/PCA-rGO/SPCEs, in presence of N_2_H_4_ and 4-NP, respectively, in the concentration range of 20–1200 µM (Figure 3A,B), to evaluate the analytical performance of the AuNPs/PCA-rGO/SPCEs and obtain calibration curves (Figure 3C,D).

The current response of the AuNPs/PCA-rGO/SPCEs in the standard solutions of N_2_H_4_ and 4-NP (Figure 3A,B) shows a linear relationship (y = (a ± b)x + c ± d) over the investigated concentration range, with a correlation coefficient of r^2^ = x, and exhibits two different slopes depending on the analyte concentration range (Figure 3C,D). This result allows us to infer the occurrence of two different electrocatalytic kinetic processes caused by a change in the electrode surface chemistry occurring during the red/ox processes. At low concentrations, the electrocatalytic process is controlled by the adsorption of the analyte on the electrode active sites and the sensitivity is high. On the contrary, at high concentrations, a partial saturation of the electrode active sites is expected to slow down the activation of the analyte molecules to the red/ox process, becoming the rate-determining step that induces a decrease in sensitivity [46].

Sensitivity (S), the limit of detection (LOD), the limit of quantification (LOQ), %RSD of repeatability, %RSD of reproducibility, and storage stability were determined to evaluate the performance of the AuNPs/PCA-rGO/SPCEs in the detection of N_2_H_4_ and 4-NP (Table 1).

The LODs of the investigated species estimated by using the proposed AuNPs/PCA-rGO/SPCEs were found lower and in line with the lowest values recently reported for similar state-of-the-art AuNPs/graphene nanocomposites synthesized by different approaches and deposited onto different electrodes (i.e., glassy carbon electrodes (GCEs)) [21,22,23,24,25,26,27,28] (Table S1), confirming their high electrocatalytic activity for N_2_H_4_ and 4-NP analysis. It is worth noticing that with respect to state-of-the-art glassy carbon electrodes (GCEs) based sensors, our approach takes advantage of the high sensitivity and low LODs typical of the SPCEs, from their suitability for on-site use, where portable and disposable electrodes are highly demanding, and from their low cost, being low volumes of samples necessary for the analyses.

Repeatability was assessed by measuring the electrocatalytic current of N_2_H_4_ and 4-NP by chronoamperometry, nine times in one day by using the same hybrid platform (Figure S3), and a satisfactory %RSD, namely 3.1 and 3.4, respectively (Table 1) were determined, as also indicated by the histograms reported in Figures S4A and S5A. Reproducibility in the detection of N_2_H_4_ and 4-NP was investigated with nine different hybrid platforms (Figure S6), and low %RSD, namely of 3.7 and 3.3, respectively, were determined (Table 1, Figures S4B and S5B). Finally, to investigate the storage stability, nine AuNPs/PCA-rGO/SPCEs were stored at 4 °C for one month, and during this time, the currents of oxidation of N_2_H_4_ and reduction of 4-NP (Figure S7) were measured every week, showing almost stable current values (Table 1, Figures S4C and S5C).

##### Interference Measurements 

Possible components of the matrix may affect the analysis of real samples, and the analytical parameters of the method, including LOD, LOQ, repeatability, and reproducibility, thus causing inaccuracy in the measure. Therefore, to investigate the role of interferent species in the detection of N_2_H_4_ and 4-NP, DPV analysis was performed by spiking 0.1 M PBS solutions at pH 7.4, 0.1 mM in N_2_H_4_ and 0.1 mM in 4-NP, respectively, with 10 mM interfering species. Citric acid, uric acid, ethanol, and glucose were added as interferents for the N_2_H_4_ detection, while catechol, hydroquinone and 2,4-dinitrobenzene, were added for the 4-NP determination (Figure S8). The results show that the interferent species added in a 100-fold higher concentration do not significantly affect the current intensity (Figure S8), showing a %RSD of 3.4 % (Figures S4D and S5D) for both the analytes, thus confirming the high selectivity of the modified electrodes to both analytes.

##### Analysis of N_2_H_4_ and 4-NP in Real Water Samples

To assess the performance and validate the proposed nanostructured platforms in the analysis of complex real samples, river and tap water samples were analyzed by chronoamperometry, and, as a comparison, by HPLC, the technique routinely used in quality control laboratories for detecting N_2_H_4_ and 4-NP [47] (Table 2). These experiments were performed by the standard addition method, by analyzing three samples added by three known concentrations of the target analytes, between 500–800 µM range, and the results were reported as mean values obtained by analyzing three aliquots of the same sample (Table 2). 

The chronoamperograms recorded for the N_2_H_4_ oxidation and the 4-NP reduction in river and tap water samples are reported in (Figure S9) at the concentrations of 600 µM and 800 µM, respectively. The recovery rates reported in Table 2 point out the reliability of the modified electrodes for the detection of the investigated compounds in the real water samples.

#### 2.3.2. Electroanalytical Investigation of Hg^2+^ at the IIP/AuNPs/PCA-rGO/SPCEs

Hg^2+^ ion imprinted polymerization of curcumin (CM) at the AuNPs/PCA-rGO/SPCEs surface was performed in potentiodynamic mode, by applying multiple potential cycling, as reported in Materials and Methods. The Hg^2+^ imprinted electropolymerized polyCM film is expected to bind, by coordination, the AuNPs surface of the hybrid modified electrodes by means of its keto groups [48]. 

Detection of Hg^2+^ was performed by monitoring the CM oxidation current at the IIP/AuNPs/PCA-rGO/SPCEs, relative to the variation of its concentration, by DPV. The addition of Hg^2+^ leads to a decrease in the oxidation peak current of CM at 0.04 V (vs. pseudo-Ag/AgCl) with the simultaneous appearance of a new peak at 0.6 V (vs. pseudo-Ag/AgCl) (Figure S10A), that is due to the oxidation of the CM, that is involved, by its keto groups, in a complex with Hg^2+^ (Hg^2+^:CM complex) [35,49]. 

To optimize the Hg^2+^ ion imprinted polymerization of CM at the AuNPs/PCA-RGO/SPCEs, different conditions of pH, number of CV scans, and Hg^2+^:CM molar ratio, were systematically investigated because such parameters affect the quality and thickness of the electropolymerized film, as well as its response to the Hg^2+^ ions [34]. The results showed that the highest oxidation current of the Hg^2+^:CM complex occurs at pH 7.4, with 20 CV scans and for the Hg^2+^:CM molar ratio of 1:4 (Figure S11). 

**Figure 4:**
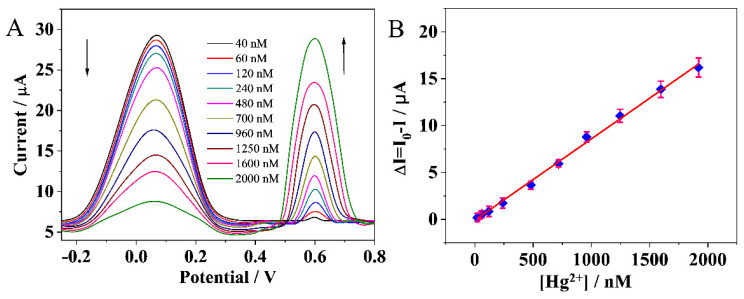
(**A**) DPVs and (**B**) corresponding calibration plot of Hg^2+^ at the IIP/AuNPs/PCA-rGO/SPCEs recorded in the potential range of −0.3 V and +0.8 V (vs. pseudo-Ag/AgCl), with 0.05 s modulation time, 0.2 s interval time, 60 mV modulation amplitude, 10.5 mV step potential, and 50 mV s^₋1^ scan rate, in 0.1 M PBS (pH 7.4) solution, after consecutive additions of Hg^2+^ from 0.04 to 2 µM.

The IIP/AuNPs/PCA-rGO/SPCEs showed good sensitivity for the electroanalytical detection of the Hg^2+^ ions by DPV because the increase in the Hg^2+^ concentration provided the decrease in the oxidation current of the non-complexed CM and the increase in the oxidation current of the Hg^2+^:CM complex (Figure 4A) [35]. Conversely, the NIP/AuNPs/PCA-rGO/SPCEs control electrode shows a negligible decrease in the oxidation current of CM upon the addition of Hg^2+^ ions (Figure S10B), attesting the effectiveness of the IIP/AuNPs/PCA-rGO/SPCEs in the determination of Hg^2+^. 

The analytical performance of the IIP/AuNPs/PCA-rGO/SPCEs was tested by DPV in 0.04–2 µM Hg^2+^ PBS buffer (pH 7.4) solutions. Figure 4B shows that the relationship between the concentration of Hg^2+^ and the oxidation current of CM (ΔI) is linear in the investigated concentration range and a LOD of 0.62 nM was found. This value is among the lowest reported in the literature for similar nanocomposites prepared by different strategies [10,11,12,13,14,15,16,17,18,19,20,21,22,23,24,25,26,27,28,29,30,31,32,33] (Table S2).

Repeatability of the IIP/AuNPs/PCA-rGO/SPCEs was assessed by measuring nine times on the same day and with the same platform, by chronoamperometry, the oxidation current of CM for the detection of 1.25 µM Hg^2+^ (Figure S12A), and the results demonstrate a good %RSD of 3.8% (Figure S13A). Reproducibility and storage stability of the IIP/AuNPs/PCA-rGO/SPCEs were tested by chronoamperometry using nine platforms (Figure S12B). The %RSD of the reproducibility was found ca. 3.2% (Figure S13B), whilst the electrochemical response for Hg^2+^ detection retained ca. 94.2% of the initial value after 30 days of storage of the electrodes at 4°C (Figure S13C), indicating their good stability. 

Selectivity was investigated by applying the IIP/AuNPs/PCA-rGO/SPCEs in the detection of possible interfering ions, such as Ca^2+^, NH_4_^+^, Na^+^, K^+^, Pb^2+^, Zn^2+^, Fe^2+^, and Ni^2+^ at a concentration 100-fold higher than the Hg^2+^ concentration (Figure S12D). In such a condition, the ΔI value of Hg^2+^ ions was found 10.94 µA, much higher than that obtained for the other investigated metal ions, with %RSD of 3.1% (Figure S13D), assessing high selectivity of the IIP electrodes towards Hg^2+^ cations due to their binding at the O,O′-donor sites of the CM tautomeric keto form [50].

Finally, to validate the method, the IIP/AuNPs/PCA-rGO/SPCEs were applied to detect, by chronoamperometry, Hg^2+^ ions in the river and tap water spiked samples (Figure S14).

The values of concentrations determined were compared with those obtained by ICP, the standard laboratory technique used for Hg^2+^ analysis [51]. The results show good recovery rates (Table 3), indicating a good agreement between the results from the developed approach with the routine analysis method.

## 3. Materials and Methods

### 3.1. Reagents and Instrumentation

Commercial reduced graphene oxide (rGO) (1.6 nm thick flakes) was purchased from Graphene Supermarket. 1-pyrene carboxylic acid (PCA, 97%), n-methyl-2-pyrrolidone (NMP, 99%), tetraoctylammonium bromide (TOAB, 99%), tetrachloroauric(III) acid trihydrate (HAuCl_4_ × 3H_2_O, 99.999%), 3,4-dimethylbenzenethiol (DMBT, 98%), sodium borohydride (NaBH_4_ 99.99%), calcium chloride, ammonium chloride, sodium chloride, potassium chloride, iron(II) chloride, standard heavy metal ion solutions of Hg^2+^, Zn^2+^, Ni^+^, and Pb^2+^ at 1000 ppm in 2% nitric acid (AAS grade), curcumin (CM), hydrazine solution (N_2_H_4_, 35 wt%) in water, 4-nitrophenol (4-NP), citric acid, uric acid, glucose, ethanol, catechol, hydroquinone, 2,4-dinitrobenzene, phosphate saline buffer (PBS) tablets, ferricyanide (Fe[(CN)_6_]^3₋^) and ferrocyanide (Fe[(CN)_6_]^4₋^), toluene and methanol were purchased from Sigma-Aldrich. Heavy metal ion solutions were prepared to dilute the standard solutions with Milli-Q water (18.2 MΩ cm at 25°C, organic carbon content ≥ 4 μg L^₋1^) achieved by a Milli-Q gradient A-10 system.

TEM analyses were performed by means of a Jeol Jem-1011 microscope operating at 100 kV and equipped with a high-contrast objective lens, a W filament as an electron source with an ultimate point resolution of 0.34 nm. Images were acquired by a Quemesa Olympus CCD 11 Mp Camera. Samples for analysis were prepared by dipping 300-mesh amorphous carbon-coated Cu grids in toluene sample solutions, then leaving the solvent to evaporate. Statistical analysis of NP average size and size distribution were performed by freeware ImageJ analysis program.

Field emission scanning electron microscopy (FE-SEM) was performed by a Zeiss Sigma microscope, operating between 0–10 KV, and equipped with both an in-lens secondary electron detector, and an INCA energy dispersive spectroscopy (EDS) detector. Samples were mounted onto stainless-steel sample holders by a double-sided conductive carbon tape and grounded by silver paste.

High-performance liquid chromatography (HPLC) measurements were performed by an Agilent 1100 HPLC analyzer (California USA) using a C18 column (5 µm, 4.6 mm × 250 mm) from waters equipped with an absorption spectrophotometer.

UV–Vis and fluorescence spectra were recorded by a Fluorolog-3 fluorometer equipped with a MicroMax 384 microplate reader. ICP-MS analysis was carried out by a NexION ICPMS spectrometer.

All electrochemical measurements were performed by a Metrohm Autolab PGSTAT 302n electrochemical workstation (Herisau, Switzerland) using screen-printed carbon electrodes (SPCEs) purchased from Dropsens. SPCEs are formed by a 4 mm diameter working electrode, a carbon counter electrode, and an Ag pseudo-reference electrode. Electrochemical data were processed by Nova^®^ v1.11 software. 

### 3.2. Synthesis of the Au NPs/PCA-rGO Hybrid Nanocomposite

The hybrid nanocomposite formed by PCA functionalized rGO sheets decorated with colloidal Au NPs (Au NPs/PCA-rGO) was synthesized by using the in situ colloidal chemical approach reported in [37]. In a typical synthesis, 5 mL of a 3 mg mL^₋1^ PCA-rGO solution in NMP was added to 35 mL of a 0.068 g mL^₋1^ TOAB toluene solution and left to stir for 30 min. Then, 15 mL of a 0.3 M HAuCl_4_ × 3H_2_O Milli-Q water solution was added and left to stir for 30 min. After TOAB-assisted transfer of HAuCl_4_ × 3H_2_O from water to toluene, water was removed from the reaction flask, 60 µL of DMBT was added, and the solution was left to stir for 1 h. In this process, DMBT slowly reduces Au(III) to Au(I), which is then reduced to Au(0) by addition of 25 mL of a 0.01512 g mL^₋1^ NaBH_4_ Milli-Q water solution. The whole process was performed at room temperature. Au NPs/PCA-rGO dispersions were purified with addition of methanol in excess, followed by three cycles of centrifugation (3000 rpm, 10 min), and finally dispersed in 7 mL toluene to achieve the concentration of 6.4 × 10^₋2^ M in Au NPs and 2 mg mL^₋1^ in PCA-rGO. A separation procedure was performed, centrifugating three times the toluene dispersion of the as-synthesized Au NPs/PCA-rGO added with methanol (3300 rpm for 30 min), to separate the Au NPs/PCA-rGO hybrid nanocomposite from the DMBT-coated Au NPs homonucleated in the synthesis mixture [37]. The isolated hybrid nanocomposite pellet was finally re-dispersed in 2.5 mL of toluene for further investigation. 

### 3.3. Preparation of the AuNPs/PCA-rGO Modified SPCEs (AuNPs/PCA-rGO/SPCEs) 

**Figure 5:**
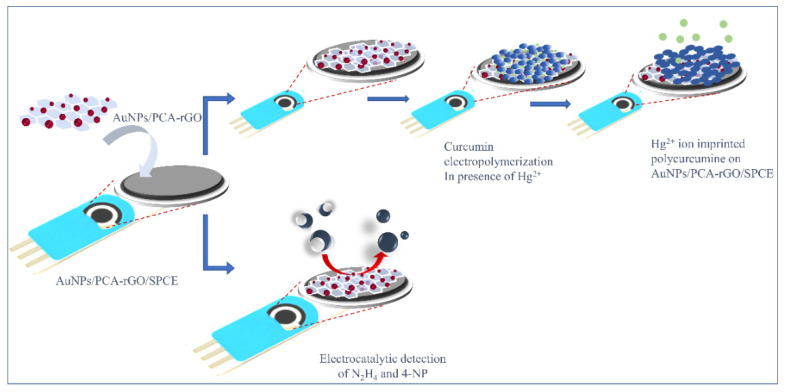
Scheme of the sensing approaches investigated in the work for the electrocatalytic detection of N_2_H_4_ and 4-NP by the AuNPs/PCA-rGO/SPCEs, and for the determination of Hg^2+^ ions by the IIP/AuNPs/PCA-rGO/SPCEs.

SPCEs were coated with the AuNPs/PCA-rGO hybrid nanocomposite (AuNPs/PCA-rGO/SPCEs) for the electrocatalytic detection of N_2_H_4_ and 4-NP in aqueous samples (Figure 1). The AuNPs/PCA-rGO/SPCEs were modified by Hg^2+^ ion imprinted electropolymerized films of polycurcumin (IIP/AuNPs/PCA-rGO/SPCEs) for the determination of Hg^2+^ ions in aqueous solutions (Figure 5).

#### 3.3.1. AuNPs/PCA-rGO/SPCEs for Electrocatalytic Detection of N_2_H_4_ and 4-NP

For the electrocatalytic detection of N_2_H_4_ and 4-NP, SPCEs preliminarily underwent to 50 cyclic voltammetry (CV) scans in the −1.5–1.5 V potential range, at the scan rate of 50 mV s^₋1^, in 0.1 M PBS buffer solution at pH 7.4. Then, the SPCEs were modified by drop-casting 5 µL of a 1:10 diluted toluene solution of the Au NPs/PCA-rGO hybrid nanocomposite.

#### 3.3.2. Hg^2+^ Imprinted Polycurcumin Modified AuNPs/PCA-rGO/SPCEs for the Electroanalytical Detection of Hg^2+^

For the electroanalytical detection of Hg^2+^ ions, Hg^2+^ imprinted polycurcumin modified AuNPs/PCA-rGO/SPCEs (IIP/AuNPs/PCA-rGO/SPCEs) were prepared by in situ electropolymerization of curcumin (CM), from a 1 mM Hg^2+^, 5 mM CM and 0.1 M PBS buffer solution, at pH 7.4, where Hg^2+^ ion is the template and CM is the functional monomer. The electropolymerization was carried out by applying 20 CV scans in the −0.3 V to +0.8 V (vs. pseudo-Ag/AgCl) range, at the scan rate of 50 mV s^−1^. To leach imprinted Hg^2+^ ions out polyCM films, CV scans in the −0.8 V to +0.8 V (vs. pseudo-Ag/AgCl) range were performed at the electrodes soaked in 0.1 M HCl solutions, until a stable oxidation peak of CM was observed. 

Non-imprinted polyCM AuNPs/PCA-rGO/SPCEs (NIP/AuNPs/PCA-rGO/SPCEs) were also prepared as control electrodes, performing the in situ electropolymerization of CM in absence of Hg^2+^ ions.

### 3.4. Electrochemical Characterization of the Nanostructured Platforms

Cyclic voltammetry (CV) and electrochemical impedance spectroscopy (EIS) measurements were performed to characterize the SCPEs, as bare and modified with PCA-rGO, AuNPs/PCA-rGO, and IIP/AuNPs/PCA-rGO, in 0.01 M PBS buffer solution (pH 7.4), 0.1 M in KCl and 5 mM in Fe[(CN)_6_]^3₋/4₋^.

EIS measurements were reported in form of complex plane diagrams (i.e., Nyquist plots), and Randles equivalent circuits were used to fit the acquired data by using the Nova^®^ v1.11 software. Charge transfer resistance (R_et_) was taken as analytical signal. 

Electroactive surface area (A_ele_) was estimated by using Randles–Sevcik equation for a quasi-reversible system, as:

I_ap_ = (2.69 × 10^5^) A_ele_ × C × D^1/2^ × n^3/2^ × v^1/2^ (1)

The heterogeneous electron transfer rate constant (k_0_) and the electron transfer resistance (R_et_) were estimated by using the following:

k_0_ = R/(n² × F² × A_ele_ × C × R_et_) (2)

In Equations (1) and (2), I_ap_ is the anodic peak current, D is the diffusion coefficient of [Fe(CN)_6_]^4₋^ in solution (6.5 × 10^₋6^ cm^2^ s^₋1^), R is the universal gas constant, F is the Faraday’s constant, n is the number of electrons transferred in the redox reaction, v is the potential scan rate (V s^₋1^) and C is the [Fe(CN)_6_]^4₋^ concentration in the bulk solution (mol cm^₋3^). 

Electrocatalytic rate constants (K_cat_) were estimated by chronoamperometry in 0.1 M PBS buffer solutions (pH 7.4), 0.1–1 mM in N_2_H_4_ and 4-NP, respectively, collected at +0.15 V and −0.71 V, respectively [52], according to Cottrell equation, as:

I_cat_/I_0_ = (π × K_cat_ × C × t)^1/2^ (3)

where I_cat_ and I_0_ are the currents, respectively, in absence and in presence of the analyte at the concentration C, K_cat_ is electrocatalytic rate constant and t is the measurement time.

### 3.5. Electroanalytical Application of the Sensing Platforms 

4-NP and N_2_H_4_ were detected by differential pulse voltammetry (DPV) by casting onto SPCEs, 50 µL of a 0.1 M PBS buffer solution (pH 7.4) added by N_2_H_4_ and 4-NP in the 20–1200 µM concentration range, respectively, with 0.05 s modulation time, 0.2 s interval time, 60 mV modulation amplitude, 10.5 mV step potential, and 50 mV s^₋1^ scan rate. 

Hg^2+^ ions were detected by DPV by monitoring CM oxidation peak current as a function of Hg^2+^ ions concentration at 100 mV potential amplitude, 2 mV step potential, 200 ms pulse time and 0.01 V s^₋1^. Before each electrochemical measurement, the IIP/AuNPs/PCA-rGO/SPCEs were exposed for 15 min to 0.04–2 µM Hg^2+^ solutions, achieved by consecutive additions of Hg^2+^, and then carefully rinsed with water. Changes in CM oxidation peak current were estimated as:

ΔI = I_0_ − I_n_ (4)

where I_0_ and I_n_ are the current intensities of the CM oxidation at t = 0 and after exposure to Hg^2+^ ions solutions at the n concentration, respectively. 

Oxidation current of CM reverts to its initial value I_0_, after regeneration of the IIP/AuNPs/PCA-rGO/SPCEs by leaching Hg^2+^ ions out the polyCM film. 

Calibration plots were adjusted to a linear model function (y = ax + b) by the weighted linear least squares method by using Origin Pro 2018 (Origin Lab Corporation, Northampton, MA, USA) and w = 1/σi^2^ as weight. 

Limit of detection (LOD) was estimated as:

LOD = 3.3 (s_y/x_/S) (5)

where s_y/x_ is the residual standard deviation and S is the slope of the calibration plot (calibration sensitivity) [53].

The limit of quantification (LOQ) was estimated as:

LOQ = 10 (s_y/x_/S) (6)

The effect of interferent species in the analyte detection was investigated by DPV in the case of N_2_H_4_ and 4-NP, and by chronoamperometry for Hg^2+^. Citric acid, uric acid, ethanol, and glucose were considered as the interferents of N_2_H_4_, catechol, hydroquinone, and 2,4-dinitrobenzene of 4-NP and Ca^2+^, NH_4_^+^, Na^+^, K^+^, Pb^2+^, Zn^2+^, Fe^2+^, and Ni^2+^ of Hg^2+^. 

An amount of 0.1 M PBS solutions at pH 7.4, containing 0.1 mM N_2_H_4_ and 0.1 mM 4-NP, respectively, were spiked with the relative interfering species at the concentration of 10 mM. In the case of Hg^2+^, 0.1 M PBS solutions at pH 7.4, containing 1.25 µM Hg^2+^, were spiked with the interfering ions at the concentration of 125 µM.

For the analyses in real matrices, the Tunis tap water and the Majerda river water were filtered by a 0.2 µm PTFE filter membrane to remove suspended particles.

The measurements were performed by the standard addition method by analyzing three samples spiked by N_2_H_4_ and 4-NP in the concentrations range of 500–800 µM, respectively, and by Hg^2+^, in the concentration range of 0.3–0.7 µM. The relative results were reported as mean values obtained by analyzing three aliquots of the same sample.

All the electrochemical results were reported as mean values obtained by analyzing in triplicate the same sample. A linear regression analysis was performed using “errors-in-variables regression methods”, considering errors in both variables (X, Y). 

The obtained results were compared with those achieved by means of high-performance liquid chromatography (HPLC), as an established analytical method for the quantification of N_2_H_4_ and 4-NP in water [47], and by inductively coupled plasma mass spectrometry (ICP) for Hg^2+^ ions [51].

## 4. Conclusions 

Nanostructured platforms formed by disposable screen-printed carbon electrodes (SPCEs), modified by a hybrid nanocomposite based on 1-pyrene-carboxylic acid functionalized RGO sheets, the surface decorated with colloidal Au NPs (AuNPs/PCA-rGO/SPCEs), were tested for the detection of N_2_H_4_, 4-nitrophenol (4-NP) and Hg^2+^, among the most toxic threats of water. Detection of Hg^2+^ was carried out after Hg^2+^ ion imprinted polymerization (IIP) of curcumin at the AuNPs/PCA-rGO/SPCEs.

The AuNPs/PCA-rGO/SPCEs showed an electrocatalytic activity higher than that of the PCA-rGO/SPCEs, which can be explained by the higher electroactive surface area and higher reversibility in the red/ox processes due to the PCA molecules, which act as electrical contact, soldering adjacent rGO sheets and as an electron coupling agent in mediating Au NPs-rGO charge transfers, and due to the electrocatalytic properties of the Au NPs. The AuNPs/PCA-rGO/SPCEs showed a high sensitivity for N_2_H_4_ and 4-NP, and the IIP homologous for Hg^2+^, namely LODs of 12.4 nM, 19.5 nM, and 0.62 nM were found for N_2_H_4_, 4-NP and Hg^2+^, respectively, in line with the lowest reported for similar state-of-the-art AuNPs/graphene nanocomposites, with relatively good %RSD of repeatability and of reproducibility and good storage stability. The modified electrodes show high selectivity with %RSD of 3.5% for N_2_H_4_ and 4-NP, and of 3.1% for Hg^2+^, after the addition of interferent species in a concentration 100-fold higher than that of the analyte. Recovery rates between 102.4–103.7% for the detection of N_2_H_4_ in real water samples, 103.2% for 4-NP, and between 97.2–103.7% for Hg^2+^, assessed feasibility of the fabricated platforms for the successful detection of such threats in real samples opening the venue to their use for the determination of other hazardous species. 

## Figures and Tables

**Table 1:** Sensitivity (S), limit of detection (LOD), limit of quantification (LOQ), and %RSD of repeatability and reproducibility and storage stability of the AuNPs/PCA-rGO/SPCEs towards 0.6 mM N_2_H_4_ and 0.6 mM 4-NP, respectively.

	N_2_H_4_	4-NP
Sensitivity/µA µM^−1^	0.13	0.12
LOD/nM	12.4	19.5
LOQ/ppm	1.3	8.9
%RSD of repeatability	3.1	3.4
%RSD of reproducibility	3.7	3.3
Storage stability	Decrease by 7.2%	Decrease by 7.1%

**Table 2:** Quantification of N_2_H_4_ and 4-NP in river and tap water samples. (*) refers to the HPLC analysis of N_2_H_4_ and 4-NP.

Samples	Analytes	Standard Concentration (mM)	Concentration Determined by Chronoamperometry (mM)	%RSD	Apparent Recovery%	Concentration Determined by Conventional Techniques * (mM)
RIVER WATER	4-NP	500	512.7 ± 3.4	2.9	102.5	504.2
		600	619.2 ± 2.4	3.4	103.2	600.3
		700	718.5 ± 3.7	3.2	102.6	701.3
	N_2_H_4_	500	521.3 ± 2.7	3.4	104.2	505.9
		600	624.7 ± 3.1	3.7	104.1	601.3
		700	713.6 ± 3.4	3.1	101.9	702.5
TAP WATER	4-NP	600	609.6 ± 2.9	3.7	101.6	604.7
		700	713.1 ± 3.2	3.6	101.8	703.9
		800	821.4 ± 3.4	3.9	102.6	797.3
	N_2_H_4_	600	591.0 ± 3.8	3.1	98.5	596.7
		700	709.3 ± 4.1	3.8	101.3	697.3
		800	819.1 ± 3.7	4.1	102.3	792.6

**Table 3:** Quantification of Hg^2+^ in river and tap water samples. (*) refers to the ICP analysis of Hg^2+^.

Samples	Analytes	Standard Concentration (Mm)	Concentration Determined by Chronoamperometry (Mm)	%RSD	Apparent Recovery%	Concentration Determined by Conventional Techniques * (Mm)
RIVER WATER	Hg (II)	0.3	0.3 ± 0.2	3.7	93.3	0.3
		0.4	0.4 ± 0.4	3.5	105.0	0.4
		0.5	0.5 ± 0.7	3.9	102.0	0.5
TAP WATER		0.5	0.4 ± 0.3	3.9	84.0	0.5
		0.6	0.6 ± 0.2	4.2	105.0	0.6
		0.7	0.7 ± 0.4	3.7	102.8	0.7

## Data Availability

Not applicable.

## Supplementary Materials

The following supporting information can be downloaded at: https://www.mdpi.com/article/10.3390/molecules27238490/s1, Figure S1: TEM micrographs of the PCA-rGO, as-synthesized and purified AuNPs/PCA-rGO, and of the AuNPs/PCA-rGO and DMBT-coated Au NPs isolated in the pellet and supernatant. TEM micrographs of the PCA-rGO, as-synthesized and purified AuNPs/PCA-rGO, and of the AuNPs/PCA-rGO and DMBT-coated Au NPs isolated in the pellet and supernatant. Figure S2: SEM image of PCA-rGO/SPCEs and neat SPCEs. Table S1: Comparative table of LODs of N_2_H_4_ and 4-NP sensing platforms reported in the literature. Figure S3: Chronoamperograms of N_2_H_4_ and 4-NP collected nine times at the same AuNPs/PCA-rGO/SPCE. Figure S4: Histograms of repeatability, reproducibility, storage stability, and selectivity tests for the detection of N_2_H_4_ at the AuNPs/PCA-rGO/SPCEs. Figure S5: Histograms of repeatability, reproducibility, storage stability, and selectivity tests for the detection of 4-NP at the AuNPs/PCA-rGO/SPCEs. Figure S6: Chronoamperograms of N_2_H_4_ and of 4-NP collected by nine AuNPs/PCA-rGO/SPCEs. Figure S7: DPVs recorded at the AuNPs/PCA-rGO/SPCEs added by N_2_H_4_ and 4-NP (B), respectively, collected after one, two, three, and four weeks. Figure S8: DPVs at the AuNPs/PCA-rGO/SPCEs in PBS buffer added by N_2_H_4_ and 4-NP, respectively, in presence of interfering species. Figure S9: Chronoamperograms at the AuNPs/PCA-rGO/SPCEs recorded for the detection of N_2_H_4_ and 4-NP, in river and tap water samples, respectively. Figure S10: DPVs at the IIP/AuNPs/PCA-rGO/SPCEs and NIP/AuNPs/PCA-rGO/SPCEs before and after addition of Hg^2+^. Figure S11: Optimization of the experimental parameters for the manufacture of the IIP/AuNPs/PCA-rGO/SPCEs. Figure S12: Chronoamperograms of repeatability, reproducibility, selectivity, and storage stability at the IIP/AuNPs/PCA-rGO/SPCEs in 1.25 µM Hg^2+^ solutions. Figure S13: Histograms of repeatability, reproducibility, storage stability, and selectivity tests at the IIP/AuNPs/PCA-rGO/SPCEs in 1.25 µM Hg^2+^ solutions. Figure S14: Chronoamperograms at the IIP/AuNPs/PCA-rGO/SPCEs in Hg^2+^ spiked river and tap water samples. Table S2. Comparative table of LODs of Hg^2+^ sensing platforms reported in the literature. Ref [21,22,23,24,25,26,27,28,29,30,31,32,33,34,38].

## Sample Availability

Samples of the compounds are available from the authors.
