# Supplementary material for: Electrochemical Sensors Based on Au Nanoparticles Decorated Pyrene-Reduced Graphene Oxide for Hydrazine, 4-Nitrophenol and Hg2+ Detection in Water
Source: Molecules. 2022 Dec 2;27(23):8490. doi: 10.3390/molecules27238490 (PMC9738402; doi:10.3390/molecules27238490)
Supplement: Supplementary file 1 [file molecules-27-08490-s001.zip › molecules-2035624-supplementary.pdf]

Supplementary Materials

Electrochemical sensors based on Au Nanoparticles decorated Pyrene-Reduced Graphene Oxide for detection of hydrazine, 4-nitrophenol and Hg^{2+} hazardous pollutants in water

Alma Mejri[a], Giocomo Mandriota[b], Hamza Elfil[a], Lucia Curri[b,c], Chiara Ingrosso[*,b], Abdelmoneim Mars[a,*]

[a]Laboratory of Natural Water Treatment (LADVEN), Water Researches and Technologies Center, Techno-park Borj-Cedria, BP 273, 8020, Soliman, University of Carthage, Tunisia.

[b]CNR-IPCF Sez. Bari, c/o Dept. of Chemistry, Università degli Studi di Bari, via Orabona 4, I-70126 Bari, Italy, [c]Dept. of Chemistry, Università degli Studi di Bari, via Orabona 4, I-70126 Bari, Italy

Figure S1 shows the TEM micrographs of the PCA-rGO complex, the as-synthesized and purified AuNPs/PCA-rGO, and of the AuNPs/PCA-rGO and DMBT-coated Au NPs isolated in the pellet and supernatant, respectively after the post-synthesis separation procedure.

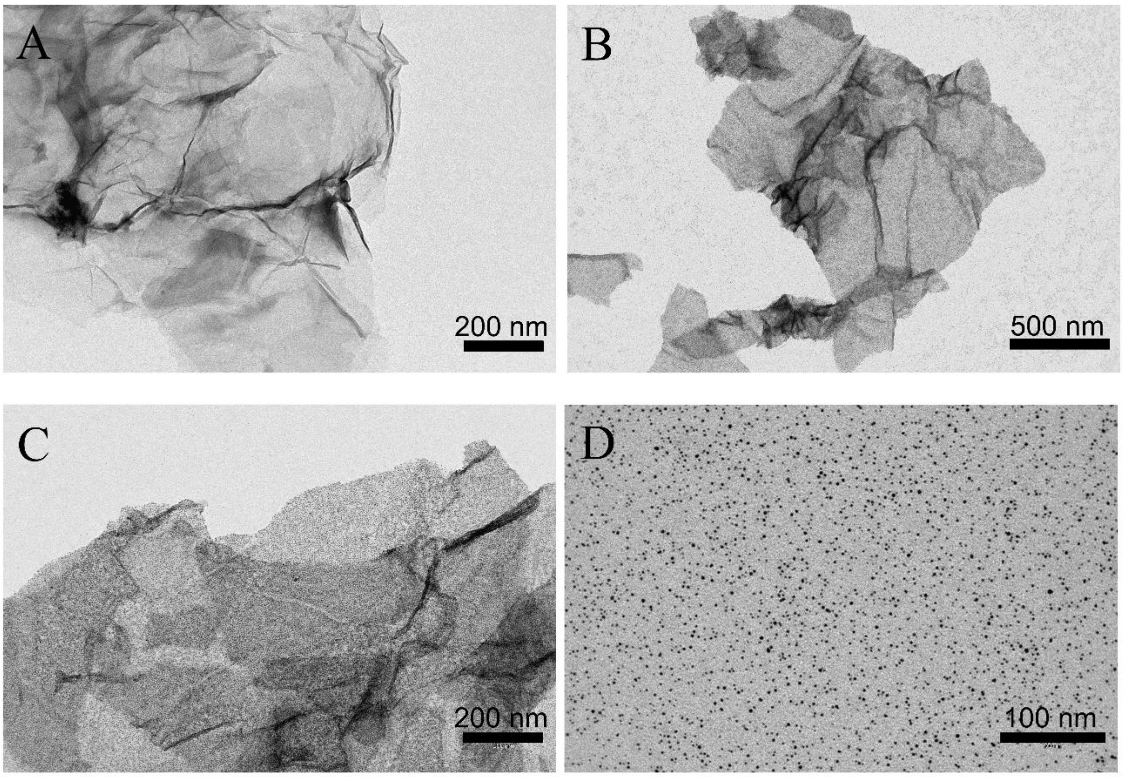

Figure S1 TEM micrographs of the PCA-rGO complex (A), as-synthesized and purified AuNPs/PCA-rGO (B), and of the AuNPs/PCA-rGO (C) and DMBT-coated Au NPs (C) isolated in the pellet and supernatant, respectively.

The TEM image of Figure S1A shows sheet-like nanostructures typical of the PCA-RGO complex. Figure S1B shows the morphology of the as-synthesized and purified AuNPs/PCA-rGO, featuring the sheets of the PCA-RGO complex, along with high image contrast, spherical in shape nano-objects, which are ascribed to the Au NPs distributing both onto the TEM grid and onto the sheets, assessing the occurrence of homonucleation of the colloidal Au NPs in the synthesis mixture.

Figure S1B shows the lack of contamination deriving from residual crystallites of TOAB, $NaBH_4$ and Au precursor, as well as the lack of low and high image contrast irregular structures which are ascribed to organic ligand molecules residual from the purification step with methanol [1], assessing the effectiveness of the purification implemented after the synthesis of the hybrid nanocomposite. Figure S1C and D show the TEM images of the Au NPs/PCA-rGO hybrid nanocomposite and of the DMBT-coated Au NPs homonucleated in the synthesis mixture, that were isolated in the pellet and in the supernatant, respectively after the separation step.

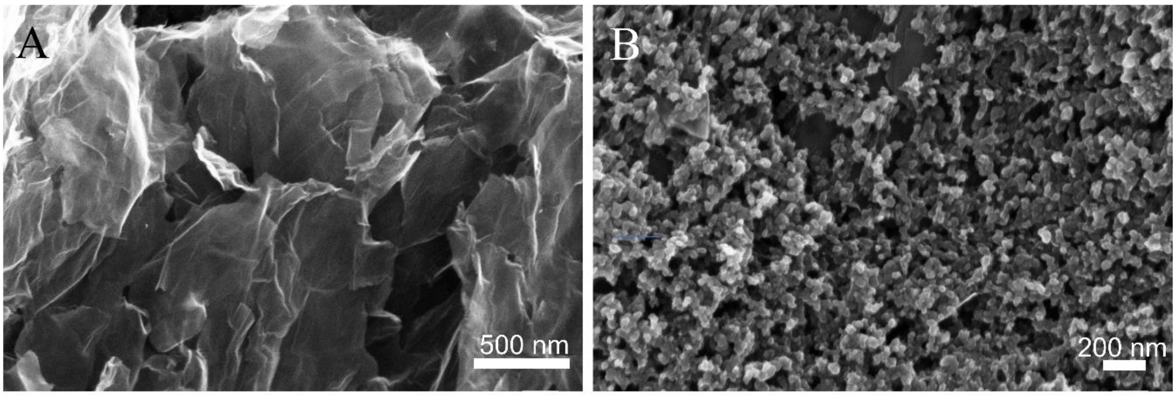

Figure S2. SEM image of (A) PCA-rGO/SPCEs and (B) bare SPCEs.

Table.S1 Comparative table of LODs of N_2H_4 and 4-NP sensing platforms reported in literature.

Electrode material	Target analyte	Linear range (mM)	Sensitivity (mA mM^{-1} cm^{-2})	LOD (nM)	%RSD	Reference
CeO_2@AuNPs/rGO/GCE	N_2H_4	30 - 3000	12.4	3	5	2
AuNPs/NPC-rGO/GCE	N_2H_4	0.05 - 880	4064	9.6	3.4	3
Au@PdNPs/rGO/GCE	N_2H_4	2 - 40	11.8	80	3.7	4
AuNPs/MWCNT/GO/GCE	N_2H_4	1-1000	0.568	380	nd	5
NG/PVP/AuNPs/SPCE	N_2H_4	2-300	1.370	70	4.8	6
AuCoNPs/PSS:PEDOT/SPCE	N_2H_4	0.5-1000	1.640	170	nd	7
AuNPs/rGO/GCE	4-NP	0.036 – 90	nd	10	nd	8
Gr/Au/GCE	4-NP	470 – 10.75 ×10^3	0.053	470	nd	9

Figure S3 reports the nine chronoamperograms of N_2H_4 and 4-NP collected at the same AuNPs/PCA-rGO/SPCEs in one day for repeatability tests.

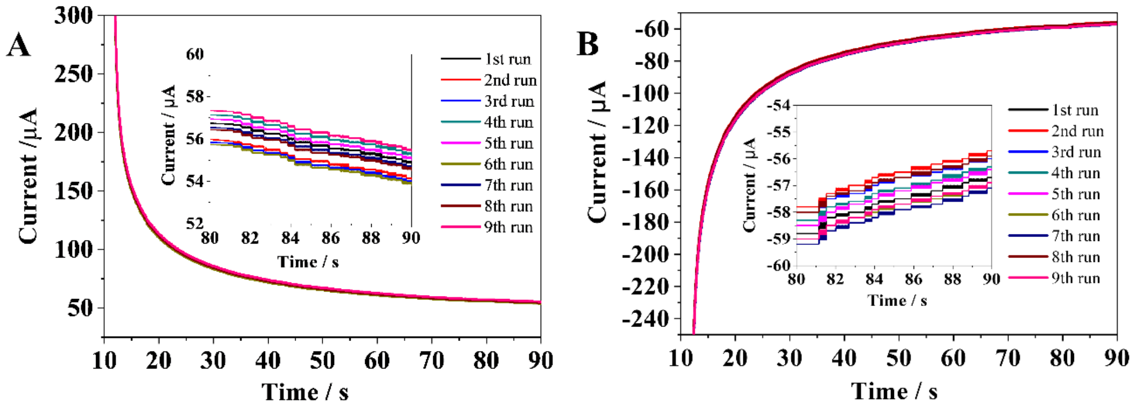

Figure S3. Chronoamperograms of (A) 0.6 mM N_2H_4 at +0.15 V (Vs pseudo-reference Ag/AgCl) and (B) 0.6 mM 4-NP at -0.71 V (Vs pseudo-reference Ag/AgCl), collected nine times at the same AuNPs/PCA-rGO/SPCE, in 0.1 M PBS buffer solutions (pH 7.4).

Figure S4 reports the histograms of repeatability, reproducibility, storage stability and selectivity tests for the detection of N_2H_4 at the AuNPs/PCA-rGO/SPCEs.

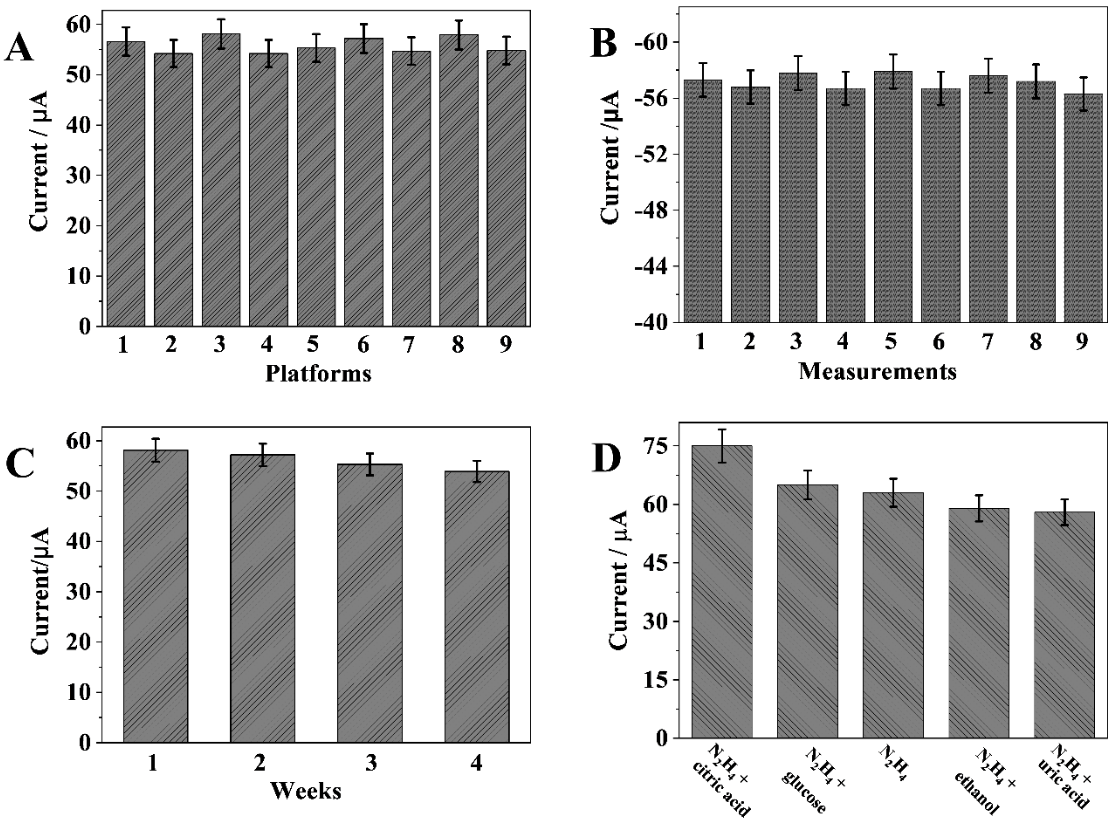

Figure S4. Histograms of repeatability (A), reproducibility (B), storage stability (C) and selectivity tests (D) for the detection of 0.6 mM N_2H_4 at the AuNPs/PCA-rGO/SPCEs.

Figure S5 reports the histograms of repeatability, reproducibility, storage stability and selectivity tests for the detection of 4-NP at the AuNPs/PCA-rGO/SPCEs.

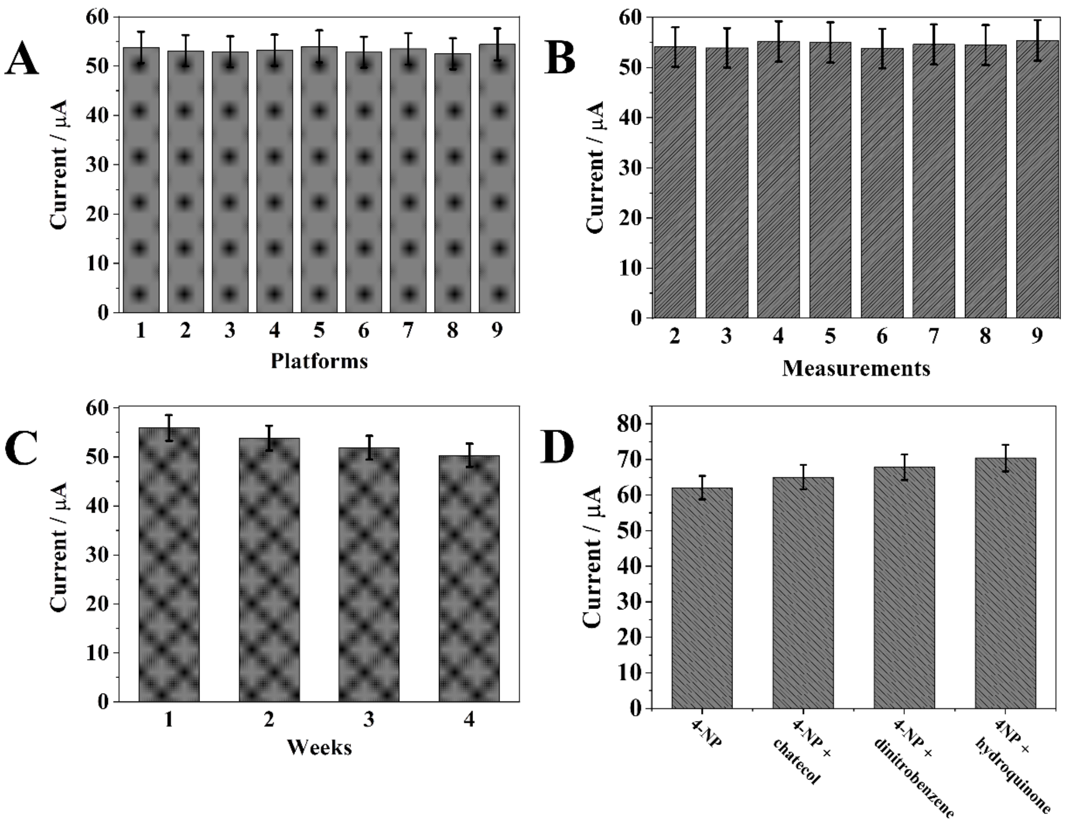

Figure S5. Histograms of repeatability (A), reproducibility (B), storage stability (C) and selectivity tests (D) for the detection of 0.6 mM 4-NP at the AuNPs/PCA-rGO/SPCEs

Figure S6 reports the chronoamperograms of N_2H_4 and 4-NP collected at nine AuNPs/PCA-rGO/SPCEs, respectively in one day for reproducibility tests.

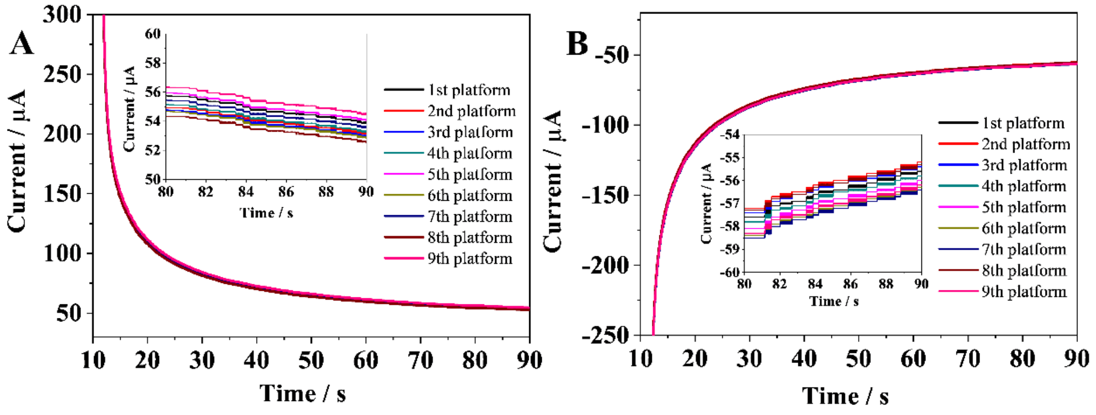

Figure S6. Chronoamperograms of (A) 0.6 mM N_2H_4 at +0.15 V (Vs pseudo-reference Ag/AgCl) and (B) of 0.6 mM 4-NP at -0.71 V (Vs pseudo-reference Ag/AgCl) collected by nine AuNPs/PCA-rGO/SPCEs, respectively in 0.1 M PBS buffer solutions (pH 7.4).

Figure S7 reports DPVs recorded at the AuNPs/PCA-rGO/SPCEs in 0.1 M PBS buffer (pH 7.4) added by N_2H_4 and 4-NP, respectively for storage stability tests.

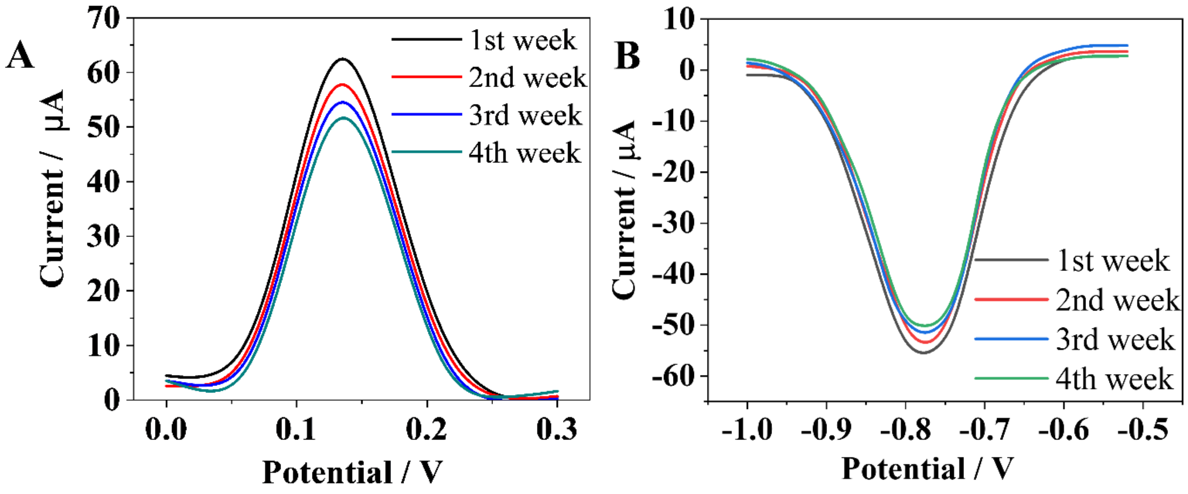

Figure S7. DPVs recorded at the AuNPs/PCA-rGO/SPCEs in 0.1 M PBS buffer (pH 7.4) added by 0.6 mM (A) N_2H_4 and (B) 4-NP, respectively, collected after one, two, three and four weeks, with 0.05 s modulation time, 0.2 s interval time, 60 mV modulation amplitude, 10.5 mV step potential and 50 mV s^{-1} scan rate.

Figure S8 reports DPV curves of aqueous samples containing N_2H_4 and 4-NP, respectively, spiked with 100-folds higher concentrated interfering species.

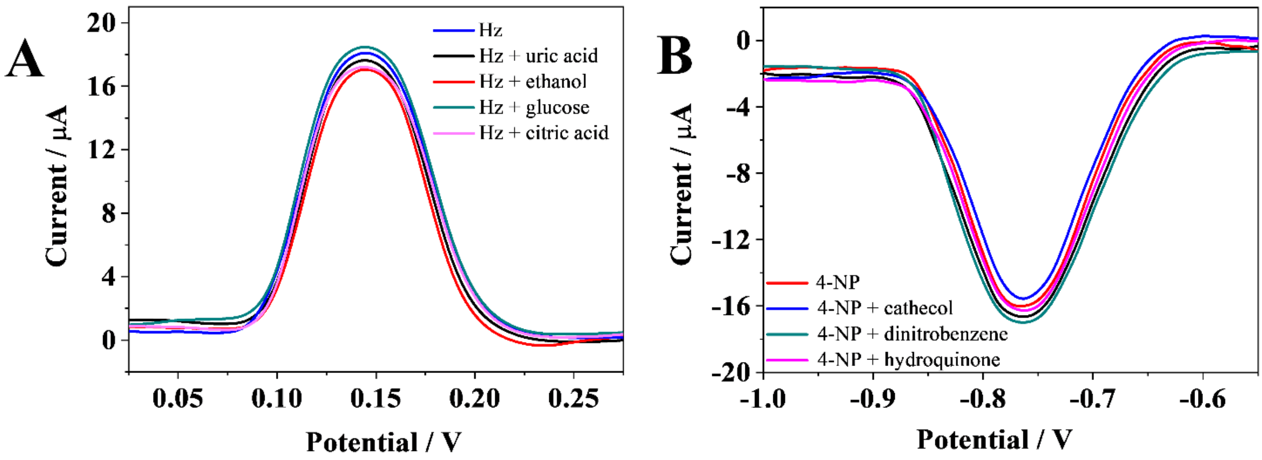

Figure S8. DPVs at the AuNPs/PCA-rGO/SPCEs in 0.1 M PBS buffer (pH 7.4) added by 0.1 mM (A) N_2H_4 and (B) 4-NP, respectively in presence of 10 mM interfering species, with 0.05 s modulation time, 0.2 s interval time, 60 mV modulation amplitude, 10.5 mV step potential and 50 mV s^{-1}scan rate.

Figure S9 reports choroamperograms recorded at the AuNPs/PCA-RGO/SPCEs for the detection of N_2H_4 and 4-NP in real water samples.

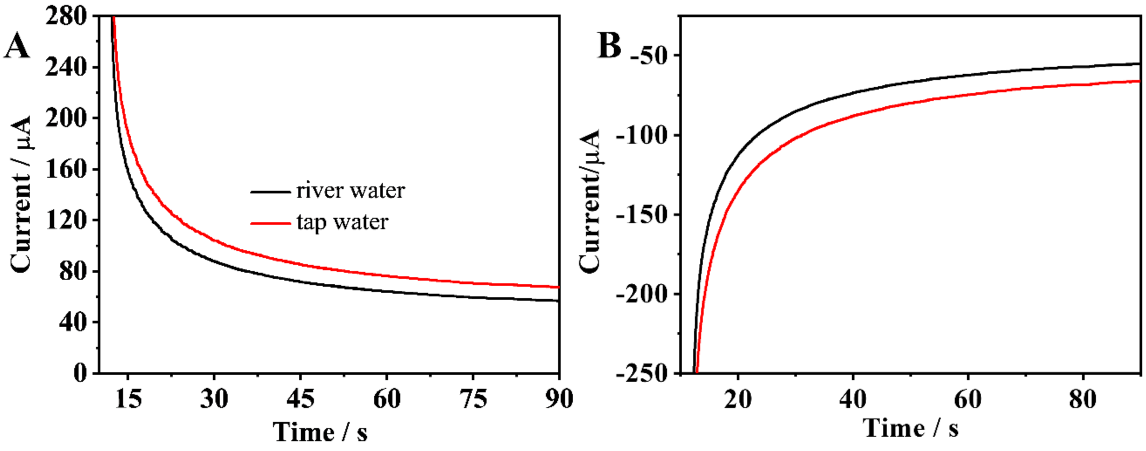

Figure S9. Chronoamperograms at the AuNPs/PCA-rGO/SPCEs recorded in the detection of (A) 600 µM N_2H_4 and (B) 800 µM 4-NP, in river and tap water samples, at 0.15 V and -0.71 V (Vs pseudo reference Ag/AgCl), respectively.

Figure S10 shows the oxidation current of CM at IIP/AuNPs/PCA-rGO/SPCEs and NIP/AuNPs/PCA-rGO/SPCEs, before and after addition of Hg^{2+}.

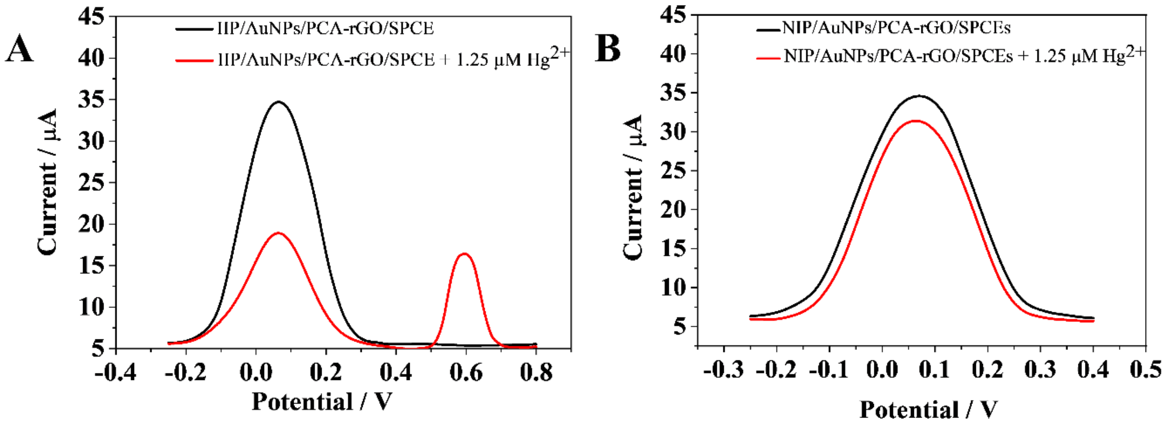

Figure S10. DPVs at the (A) IIP/AuNPs/PCA-rGO/SPCEs and (B) NIP/AuNPs/PCA-rGO/SPCEs in 0.1 M PBS buffer (pH 7.4), before and after addition of 1.25 µM Hg^{2+}, at 0.05 s modulation time, 0.2 s interval time, 60 mV modulation amplitude, 10.5 mV step potential and 50 mV s^{-1} scan rate.

Figure S11 reports the optimization of the experimental parameters used for the manufacture of the IIP/AuNPs/PCA-rGO/SPCEs.

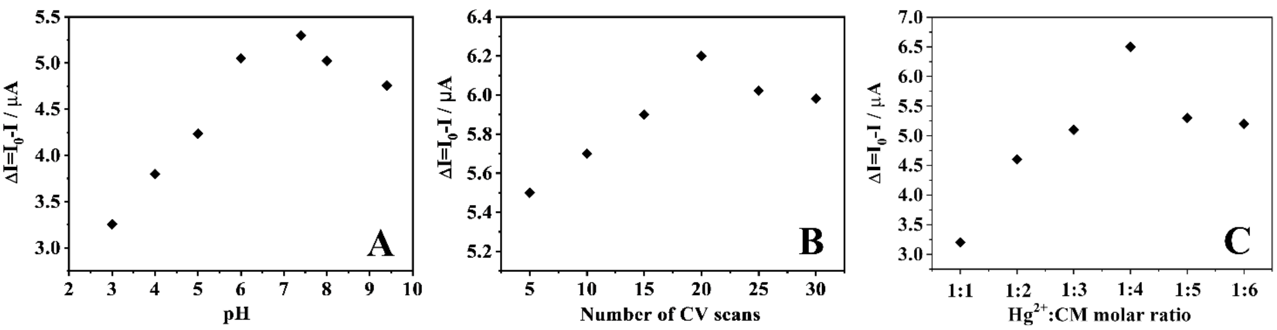

Figure S11 Optimization of the experimental parameters for the IIP/AuNPs/PCA-rGO/SPCEs manufacture: study of pH (A) and number of CV scans (B), in a 1 mM Hg^{2+} and 0.1 M PBS buffer solution, and (C) of the Hg^{2+}:CM molar ratio with 20 CV scans, in 0.1 M PBS buffer solution at pH 7.4.

Figure S12 reports the chronoamperograms of the repeatability, reproducibility, and storage stability tests of the IIP/AuNPs/PCA-rGO/SPCEs for the detection of Hg^{2+}.

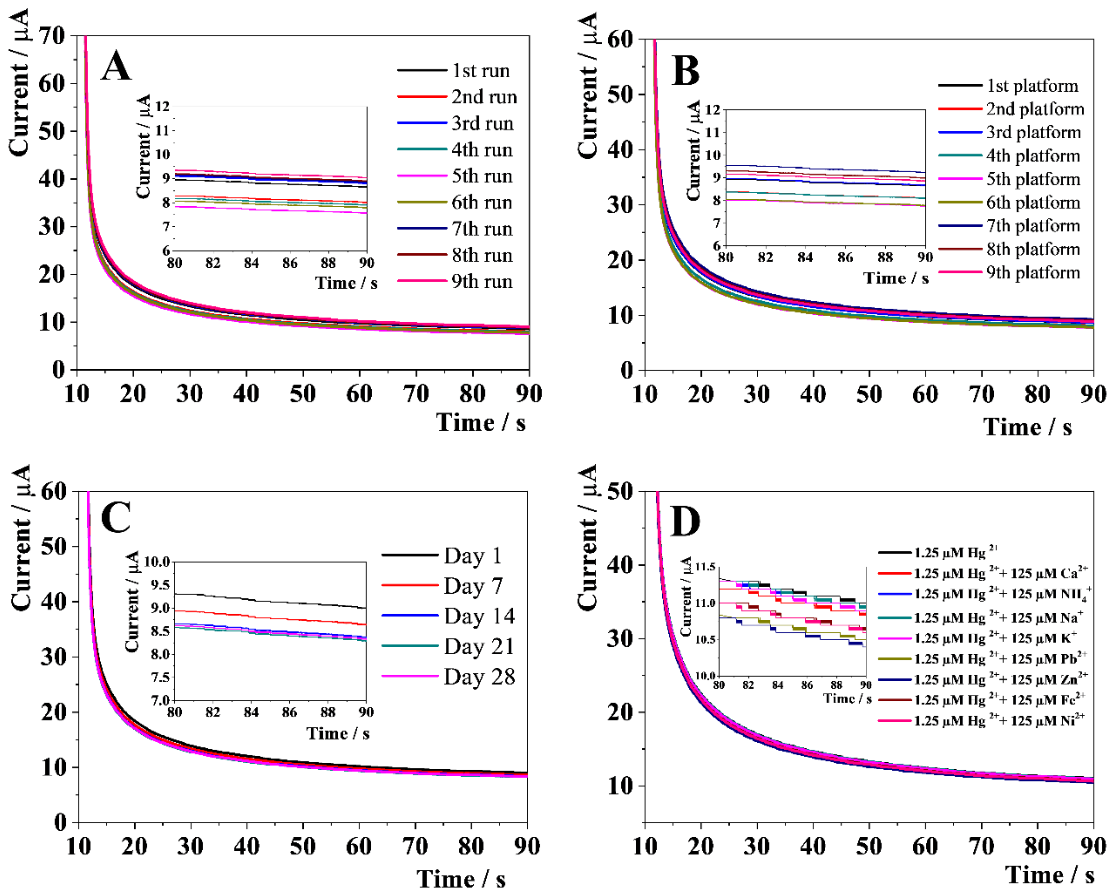

Figure S12. Chronoamperograms of repeatability (A), reproducibility (B), selectivity (C) and storage stability (D) at the IIP/AuNPs/PCA-rGO/SPCEs collected at +0.05 V (Vs pseudo Ag/AgCl) in 1.25 μM Hg^{2+} and 0.1 M PBS buffer solutions (pH 7.4).

Figure S13 reports the histograms of repeatability, reproducibility, storage stability and selectivity tests for the detection of Hg^{2+} at the AuNPs/PCA-rGO/SPCEs.

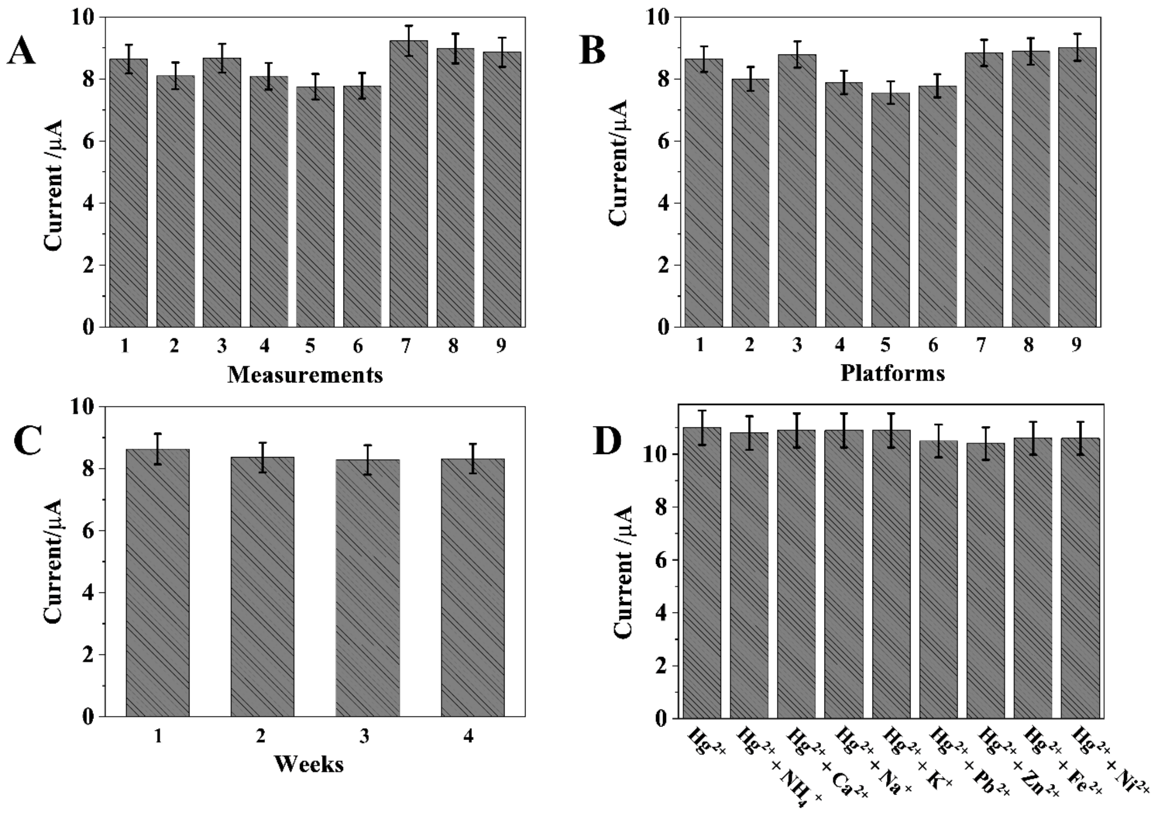

Figure S13. Histograms of repeatability (A), reproducibility (B), storage stability (C), and selectivity tests of the IIP/AuNPs/PCA-rGO/SPCEs collected at +0.05 V (Vs pseudo Ag/AgCl) in 1.25 μM Hg^{2+} and 0.1 M PBS buffer solutions (pH 7.4).

Figure S14 reports the chronoamperograms collected at the IIP/AuNPs/PCA-rGO/SPCEs in Hg^{2+} spiked river and tap water samples.

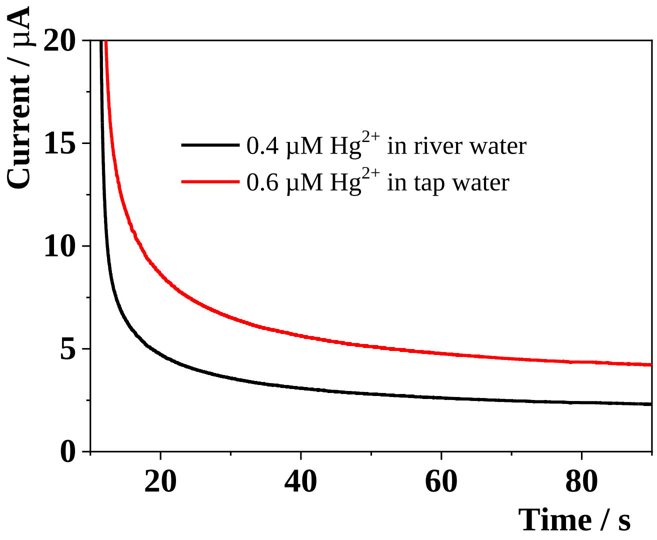

Figure S14 Chronoamperograms at the IIP/AuNPs/PCA-rGO/SPCEs in 0.4 and 0.6 µM Hg^{2+} spiked river and tap water samples, respectively at 0.05 V (Vs pseudo Ag/AgCl).

Table S2. Comparative table of LODs of Hg^{2+} sensing platforms reported in literature.

Electrode material	Linear range (mM)	Sensitivity (mA mM^{-1})	LOD (nM)	Reference
Tymine/AuNPs/rGO/GCE	5-25	0.0054	3.94	10
AuNPs/rGO/GCE	0.5-20	nd	0.06	11
AuNPs/chitosan-rGO/GCE	0.1-60	0.7080	6.00	12
AuNPs/rGO/GCE	1-10	0.5500	0.20	13
AgNWs/HPMC/chitosan/urease/SPCE	2-25	0.0054	3.94	14
Au/SPCE	1-100	nd	1.02	15

References

[1] Li, C.; Tardajos, A.P.; Wang, D.; Choukroun, D.; Daele, K. V.; Breugelmans, T.; Bals, S. A simple method to clean ligand contamination on TEM grids. Ultramicroscopy 2021, 221, 113195.

[2] H. Huang, T. Li, Y. Sun, L. Yu, C. Wang, R. Shen, W. Ye, D. Wang, Amperometric sensing of hydrazine in environmental and biological samples by using CeO_2-encapsulated gold nanoparticles on reduced graphene oxide. Microchim Acta, 2019, 186, 46.

[3] Y. Zhang, Y. Zhang, D. Zhang, L. Ding, J. Sida, S. C. Jiang, Y. Su, Confinement preparation of Au nanoparticles embedded in ZIF-67-derived N-doped porous carbon for high-performance detection of hydrazine in liquid/gas phase. Sensors and Actuators B: Chemical, 2019, 285, 607-616.

[4] S. Dutta, C. Ray, S. Mallick, S. Sarkar, A. Roy, T. Pal, Au@Pd core–shell nanoparticles-decorated reduced graphene oxide: a highly sensitive and selective platform for electrochemical detection of hydrazine. RSC Adv., 2015, 5, 51690-51700

[5] Y. J. Yang, L. Weikun, Self-Assembly of Gold Nanoparticles and Multiwalled Carbon Nanotubes on Graphene Oxide Nanosheets for Electrochemical Sensing Applications. Fullerenes, Nanotubes and Carbon Nanostructures, 2018, 12, 837-845

[6] C. Saengsookwaow, R. Rangkupan, O. Chailapakul, N. Rodthongkum, Nitrogen-Doped Graphene-Polyvinylpyrrolidone/Gold Nanoparticles Modified Electrode as a Novel Hydrazine Sensor. Sensors and actuators B, 2016, 227, 524-532

[7] D. T. Nde, H. J. Hye, J. Lee, Electrocatalytic determination of hydrazine concentrations with polyelectrolyte supported AuCo nanoparticles on carbon electrodes. Catalysis today, 2022, 403, 11-18

[8] X. X. Jiao, H. Q. Luo, N. B. Li, Fabrication of graphene–gold nanocomposites by electrochemical co-reduction and their electrocatalytic activity toward 4-nitrophenol oxidation. Journal of Electroanalytical Chemistry, 2013, 691, 83-89.

[9] Zhang, W.; Chang, J.; Chen, J.; Xu, F.; Wang, F.; Jiang, K.; Gao, Z. Graphene–Au composite sensor for electrochemical detection of para-nitrophenol. Res Chem Intermed., 2012, 38, 2443–2455.

[10] N. Wang, M. Lin, H. Dai, M. Houyi, Functionalized gold nanoparticles/reduced graphene oxide nanocomposites for ultrasensitive electrochemical sensing of mercury ions based on thymine–mercury–thymine structure. Biosens. Bioelectron., 2016, 79, 320-326.

[11] D. Shi, W. Wu, X. Li, Ultrasensitive detection of mercury (II) ions on a hybrid film of a graphene and gold nanoparticle-modified electrode. Anal. Methods, 2022, 14, 2161-2167.

[12] J. Gong, T. Zhou, D. Song, L. Zhang, Monodispersed Au nanoparticles decorated graphene as an enhanced sensing platform for ultrasensitive stripping voltammetric detection of mercury(II). Sensors and Actuators B: Chemical, 2010, 150, 491-497.

[13] N. Ramila Devi, Manickam Sasidharan, Ashok K. Sundramoorthy, Gold Nanoparticles-Thiol-Functionalized Reduced Graphene Oxide Coated Electrochemical Sensor System for Selective Detection of Mercury Ion. J. Electrochem. Soc., 2018, 165, B3046.

[14] A. Saenchoopa, S. Klangphukhiew, R. Somsub, C. Talodthaisong, R. Patramanon, J. Daduang, S. Daduang, S. Kulchat, A Disposable Electrochemical Biosensor Based on Screen-Printed Carbon Electrodes Modified with Silver Nanowires/HPMC/Chitosan/Urease for the Detection of Mercury (II) in Water. Biosensors, 2021, 11, 351.

[15] I. T. Somé, A. K. Sakira, D. Mertens, S. N. Ronkart, J. M. Kauffmann, Determination of groundwater mercury (II) content using a disposable gold modified screen printed carbon electrode. Talanta, 2016, 152, 335-340.
